# Single-pulse lithography of amorphous photonic architectures inside all-inorganic dielectric crystals

**DOI:** 10.1038/s41377-026-02253-1

**Published:** 2026-03-18

**Authors:** Zhuo Wang, Rongze Ma, Han Lin, Pengfei Zhang, Yu Lu, Feng Chen, Baohua Jia, Bo Zhang, Jianrong Qiu

**Affiliations:** 1 State Key Laboratory of Extreme Photonics and Instrumentation, College of Optical Science and Engineering, Zhejiang University, Hangzhou, 310027 China; 2 Centre for Atomaterials and Nanomanufacturing, School of Science, RMIT University, Melbourne, 3000 VIC Australia; 3 State Key Laboratory for Manufacturing System Engineering and Shaanxi Key Laboratory of Photonics Technology for Information, School of Electronic Science and Engineering, Xi’an Jiaotong University, Xi’an, 710049 China

**Keywords:** Optical materials and structures, Applied optics

## Abstract

High-efficiency light modulation within transparent substrates is critically important for advancing in-chip integrated optical technologies. However, current micro/nanophotonic platforms primarily rely on 2D surface configurations, rendering them inadequate for 3D optical design in dielectric environments. Here, we introduce a precise phase-transition technique that enables the direct lithography of highly regular amorphous units in multiple transparent dielectric crystals (lithium niobates, quartz, yttrium vanadate, etc.). This unit can be rapidly written with a single ultrafast laser pulse, exhibiting a high-purity amorphization phase transition interior structure and a regular sheet-like anisotropic spatial morphology (aspect ratio reaching 190:1). We reveal that this amorphization stems from ultrafast laser-driven anisotropic thermal deposition, achieved through the synergy of the light-induced high-density free electrons and thermal effects. Such embedded units achieve more than an order of magnitude improvement in the efficiency of nonlinear beam shaping (~3% second harmonic and ~0.1% third harmonic) and offer multiple degrees of freedom for device design. This study establishes a versatile platform for on-demand production of all-dielectric micro/nanophotonic architectures in the free space of transparent dielectrics, unlocking new avenues for 3D integrated photonics.

## Abstract (web summary)

A technique utilizing ultrafast laser-induced anisotropic thermal deposition enables precise amorphous phase transitions, achieving single-pulse 3D lithography of highly regular nonlinear photonic architectures within all-inorganic dielectric crystals.

## Graphical abstract

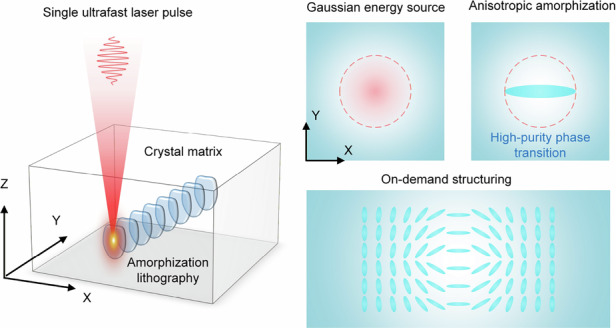

## Introduction

Phase transition, a ubiquitous phenomenon in light-matter interactions, is extensively leveraged in applications ranging from material design to micro/nanofabrication^1–7^. A prime example is photolithography^8^, a cornerstone of the semiconductor industry, which utilizes ultraviolet light to induce precisely controlled chemical phase transitions in photosensitive resists. By operating optical systems near the fundamental physical resolution limits, this technique enables the fabrication of nanoscale electronic circuits on silicon substrates^9–13^. Unlike electronic engineering, the transmission and manipulation of photons is critically dependent on inorganic transparent dielectrics, such as various glasses and crystals^14–18^. However, as key materials for next-generation information technologies, nonlinear dielectric crystals present manufacturability challenges: despite their irreplaceable optical properties, their highly stable ionic and covalent bonds and very limited linear optical absorption render conventional lithography techniques ineffective for creating embedded artificial photonic architectures.

Theoretically, every dielectric crystal possesses a corresponding amorphous phase. These amorphous phases generally exhibit significant differences in optical properties (e.g., refractive index, optical absorption, and nonlinear coefficient) relative to their crystalline counterparts^19–21^. This enables significant and non-volatile optical modulation within the crystal matrix’s high-refractive-index environment, positioning amorphous phases as a promising candidate for realizing in-chip integrated photonic functionalities. However, amorphous phase transitions rely predominantly on rapid quenching^21^, vapor deposition^22^, or high-energy particle irradiation^23^. These methods are inherently spatially non-selective and limited in resolution, thus unsuited for constructing intricate photonic structures. Up to now, achieving on-demand creation and precise manipulation of amorphous phases within dielectric crystals, with the high spatial resolution and well-defined geometries essential for photonic applications, remains a significant scientific and technological challenge.

Recently, ultrafast laser-induced non-stoichiometric material modification has been established as a versatile technique for tailoring the physicochemical properties of transparent media^24–27^. By leveraging their high peak power, ultrafast laser pulses interact with transparent materials predominantly through nonlinear optical absorption^28,29^. This mechanism enables the creation of localized high-temperature and high-pressure extreme conditions at the microscale^30^, facilitating high-precision amorphous phase transitions in dielectric crystals. However, such extreme peak power and multi-pulse synergy can simultaneously trigger multiple competing nonlinear optical effects. For instance, during cumulative pulse interactions, the light field within the focal volume tends to undergo dynamic self-organization^31–33^, leading to spontaneous intensity redistribution that significantly deviates from the intended configuration. These undesired effects generally lead to spatially inhomogeneous material modifications and unintended phase impurities, fundamentally hindering the achievement of high-efficiency optical modulation.

**Fig. 1:**
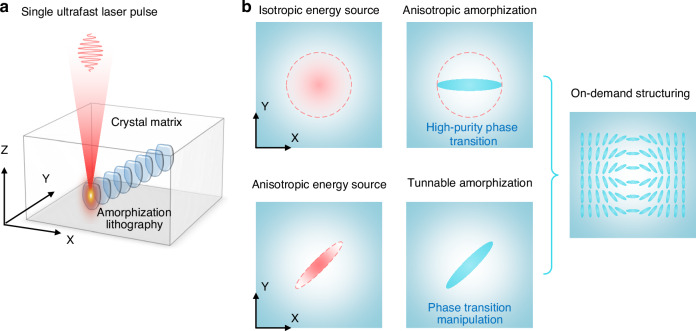
Concept of SAAL. **a** Schematic diagram of SAAL in transparent dielectric crystals. **b** Basic implementation process of SAAL using isotropic and anisotropic energy sources. Red dotted curves indicate the laser spot profile in the XY plane

In this study, using lithium niobate (LN) and quartz crystal as model dielectric systems, notable for their pronounced optical performances and anisotropic characteristics, we developed a versatile ultrafast laser-driven single-pulse anisotropic amorphization lithography (SAAL) technique that enables efficient patterning of regular and high-purity amorphization with multidimensional controllability in 3D free space (Fig. 1). By constructing fine amorphous micro-nanophotonic architectures, we achieve superior nonlinear beam shaping with second-harmonic generation (SHG) efficiency enhancements exceeding current state-of-the-art approaches by more than an order of magnitude.

## Results

**Fig. 2:**
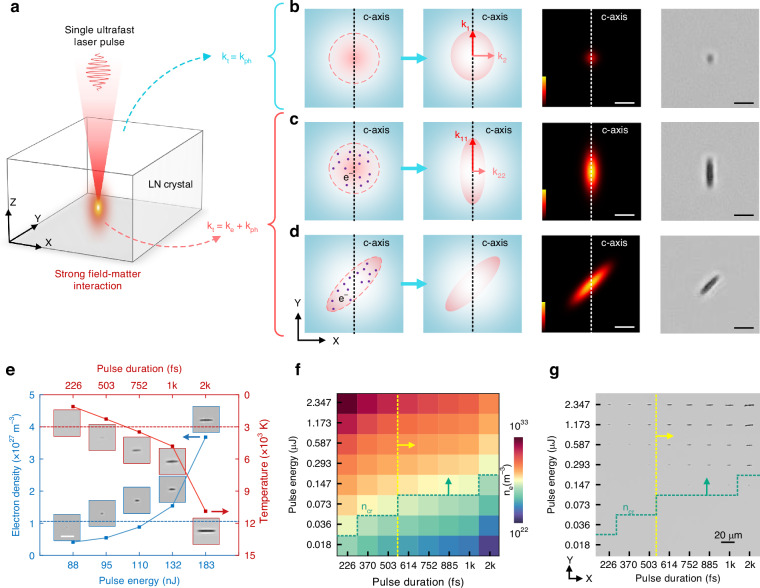
Mechanism of SAAL. **a** Schematic diagram of ultrafast laser-matter interaction in transparent dielectric crystals. Thermal deposition dominated by phonon thermal conductivity under low-energy (pulse energy = 90 nJ) Gaussian spot irradiation, where *k*_1_:*k*_2_ ≈ 1.06 (**b**); thermal deposition dominated by electronic thermal conductivity under high-energy (pulse energy = 180 nJ) Gaussian spot irradiation, where *k*_11_:*k*_22_ ≈ 10 (**c**); and thermal deposition dominated by electronic thermal conductivity under high-energy anisotropic spot irradiation, achieved with an optical slit (**d**). Conceptual diagram (left), simulated temperature field (middle), and experimentally produced structure (right). The laser pulse duration is 1 ps. Color bars: 300–1300 K. Scale bars: 2 μm. Red dotted curves indicate the ultrafast laser spot profile in the XY plane. **e** Theoretically calculated electron density and equilibrium temperature as a function of ultrafast laser pulse energy and pulse duration, respectively. Insets: experimentally produced structures. Scale bar: 5 μm. **f** Theoretical process window for SAAL. The yellow line marks a conservative pulse duration threshold for inducing significant anisotropic thermal deposition. Color bar: electron density. **g** Experimental results using the parameters in (**f**)

SAAL relies primarily on ultrafast laser-induced anisotropic thermal deposition enhanced by strong field-excited free electrons (Fig. 2a). Specifically, LN crystals belong to the trigonal system (point group 3 m)^34,35^. Their structure features distorted NbO_6_ oxygen octahedra, characterized by strongly covalent Nb-O-Nb chains along the c-axis versus a hexagonal network of octahedra in the ab-plane^36^. This inherent structural anisotropy causes phonons to propagate differently along different crystallographic directions. Theoretically, stronger bonding along the c-axis typically implies higher phonon group velocities and reduced anharmonic scattering, suggesting higher thermal conductivity parallel to the c-axis than perpendicular to it^37,38^. Nevertheless, the intrinsic phonon thermal conductivity anisotropy arising from the crystal’s structural anisotropy is insufficient to enable anisotropic thermal deposition relevant to SAAL: the measured ratio of thermal conductivity parallel versus perpendicular to the c-axis is only ~1.06^39^. Such a minor anisotropy cannot establish a pronounced anisotropic temperature field with an isotropic energy source, such as a Gaussian-typed ultrafast laser spot (Fig. 2b). In theory, achieving significant anisotropic heat deposition necessitates enhancing the crystal matrix’s thermal conductivity anisotropy by at least an order of magnitude (e.g., to a ratio of ~10). Thus, achieving SAAL solely via the intrinsic phonon thermal conductivity anisotropy of the LN crystal matrix is not feasible in practice.

Generally, the total thermal conductivity (${k}_{{\rm{t}}}$) of a system consists of two parts, including phonon thermal conductivity (${k}_{{\rm{p}}{\rm{h}}}$) and electronic thermal conductivity (${k}_{{\rm{e}}}$):

1 $${k}_{{\rm{t}}}={k}_{{\rm{ph}}}{+k}_{{\rm{e}}}$$

Although altering the phonon thermal conductivity of dielectric crystals presents inherent challenges, we propose that the thermal anisotropy of LN crystals can be significantly enhanced by injecting high-density free electrons. This enhancement arises from two key mechanisms: (1) High-density free electrons can significantly increase the electronic thermal conductivity, and (2) strong covalent bonding along the c-axis, cooperating with the significant spontaneous polarization in this direction^40,41^, provides a preferential conduction path for the excited free electrons (higher transient electron mobility)^42^, facilitating the anisotropic energy transfer from high-density hot electrons to the lattice. However, the practical implementation of this concept is constrained by the scarcity of free electrons in dielectric materials and the lack of hot electron excitation approaches, making its steady-state regime virtually unattainable.

To overcome this gap, we propose exciting high-density free electrons via ultrafast laser irradiation to create a transient metallic state in the irradiated volume. According to the Wiedemann-Franz Law, in the metallic state, the electronic thermal conductivity (${k}_{{\rm{e}}}$) and the electrical conductivity ($\sigma$) are related through the electron temperature (*T*):

2 $${k}_{{\rm{e}}}={LT}\sigma$$

where$\,L$ is the Lorenz number and $\sigma$ is proportional to electron density. Based on this, the implementation of SAAL is expected to depend critically on two factors: (1) sufficient pulse energy to ensure elevated free electron densities and electron kinetic energy, thereby enabling high electrical conductivity and electron temperature (corresponding to high ${k}_{{\rm{e}}}$); and (2) proper pulse duration to maintain photon-electron-lattice energy transfer, facilitating lattice heating (reflected by equilibrium temperature). In principle, the synergy of these two factors will drive a significant enhancement in anisotropic thermal deposition. According to the two-temperature model (Note S1), a single ultrafast laser pulse with a pulse duration of 600 fs and a pulse energy of 180 nJ can theoretically induce an electron density reaching 10^28^ m^−3^, comparable to that of metallic materials (i.e., metallic state). During the period of lattice heating, the electron temperature generally exceeds 10^6 ^K (Fig. S1a). Incorporating the Wiedemann-Franz law, we estimate that the electronic thermal conductivity can be enhanced by more than an order of magnitude stronger than the phonon thermal conductivity (Fig. S1b), becoming the dominant component of the system’s total thermal conductivity. Furthermore, the thermal flux estimation confirms that ultrafast laser-excited high-density electrons can effectively mediate energy deposition within the focal region (Note S2). In this condition, we demonstrate that even an isotropic Gaussian-typed ultrafast laser pulse can induce anisotropic material modification elongated along the c-axis (Fig. 2c), far exceeding what can be achieved by the inherent thermal conductivity anisotropy of the crystalline matrix. On this basis, we further propose that SAAL should be governed predominantly by electronic thermal conductivity. This implies that arbitrary orientation of anisotropic material modification can be achieved by artificially manipulating the profile of the ultrafast laser irradiation zone (e.g., via light field shaping) that governs the spatial distribution of high-density free electrons (Fig. 2d).

Our proposal has been firmly confirmed through systematic ultrafast laser irradiation experiments, where single Gaussian-typed ultrafast laser pulses are irradiated into a Y-cut LN crystal (with the c-axis perpendicular to the laser incident direction) to present the evolution of laser-induced structures under different irradiation conditions. As shown in Fig. 2e, theoretical calculations show that the free electron density in the laser-irradiated region increases rapidly with increasing pulse energy. Experimentally, for a pulse duration of 1 ps, when the pulse energy is below 0.11 μJ (corresponding to a free electron density less than 10^27^ m^−3^), no anisotropic structure is produced, indicating that SAAL is not activated. When the pulse energy reaches 0.13 μJ, the free electron density reaches 10^27^ m^−3^ level, causing the irradiated region to become a quasi-metallic state^43^. We demonstrate that in this case, a single ultrafast laser pulse can induce anisotropic structures elongated along the c-axis with an increased aspect ratio as the pulse energy increases. Secondly, ultrafast laser-induced anisotropic energy deposition is also strongly dependent on pulse duration. The anisotropy of the induced structures increases significantly with larger pulse durations. Experimentally, even when the pulse energy is sufficient to excite high-density free electrons, a pulse duration exceeding 0.7 ps (corresponding to an equilibrium temperature of ~3000 K) is still required to induce structures exhibiting significant anisotropy along the c-axis, confirming the necessity of a certain degree of thermal effects for SAAL. These results demonstrate that the high-density free electrons, incorporating the laser-induced thermal effects, can significantly enhance the thermal anisotropy of the irradiated region, fully consistent with our hypothesis.

Notably, SAAL using isotropic energy sources relies on intrinsic thermal anisotropy in the matrix material. For example, when a Gaussian-profile laser spot is used to irradiate a Z-cut LN crystal (c-axis parallel to the laser propagation direction), neither the light field nor the crystal matrix exhibits anisotropy, preventing SAAL activation regardless of laser parameters (Fig. S2). To overcome this limitation, we leverage the dominance of electronic thermal conductivity during the SAAL process. By constructing an anisotropic light field with parameters sufficient to excite high-density free electrons and induce substantial thermal effects, we demonstrate that SAAL-produced structural features in this condition depend exclusively on the light field’s anisotropy rather than the crystal’s c-axis orientation (Fig. S3), confirming the feasibility of on-demand SAAL. Therefore, the realization of SAAL requires three basic conditions: (1) excitation of high-density free electrons; (2) induction of sufficient thermal effects; and (3) the energy source or the matrix material exhibits structural anisotropy. Guided by the established model, we can theoretically outline the process window of SAAL in LN crystals based on the electron density and equilibrium temperature calculation (Fig. 2f), agreeing well with corresponding experimental results (Fig. 2g).

**Fig. 3:**
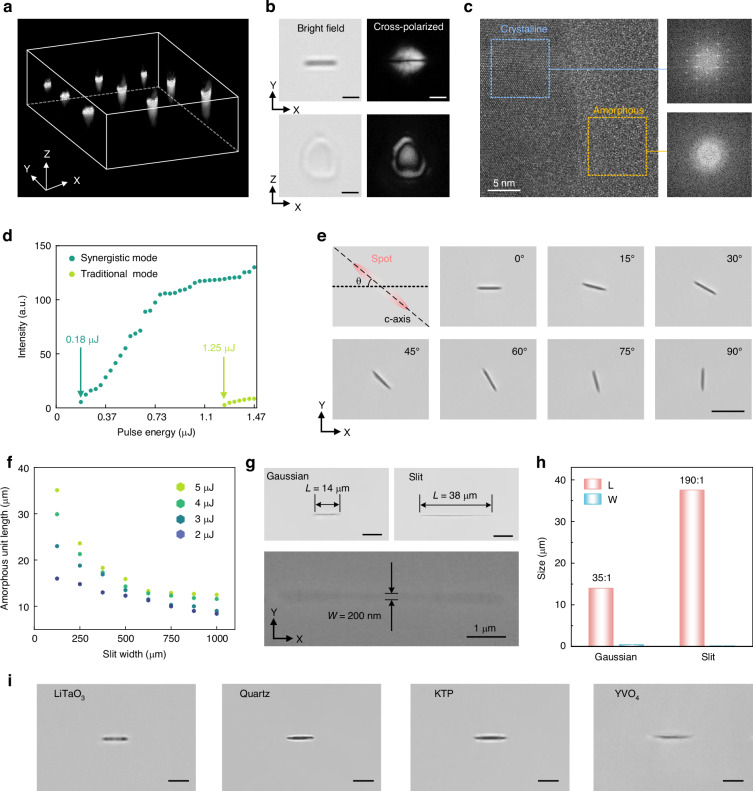
Structural characterization, manipulation, and cross-material effectiveness of SAAL. **a** Holoview 3D refractive index imaging of SAAL-produced anisotropic, sheet-like amorphous structures. **b** Bright field microscopic image and cross-polarized image of SAAL-produced amorphous structures in the XY and XZ planes. Scale bars: 2 μm. **c** HRTEM imaging of the amorphization phase-transition interface at the boundary of the SAAL-produced amorphous structures. Insets: fast Fourier transform (FFT) images of the dotted areas. **d** Birefringence intensity as a function of pulse energy under different SAAL processing modes. **e** Orientation manipulation of SAAL-induced amorphous units by using anisotropic energy sources. Scale bar: 5 μm. **f** Feature size manipulation of SAAL-induced amorphous units by tuning silt width and pulse energy. **g** Optical microscopic images of amorphous units produced by Gaussian and slit-shaped laser spots (upper) and SEM image of an amorphous unit (lower). Scale bars: 10 μm. **h** Aspect ratio comparison of amorphous units produced by Gaussian and slit-shaped laser spots. **i** Cross-material effectiveness of SAAL. All presented amorphous units are induced by a Gaussian-typed beam spot. Scale bars: 5 μm

We demonstrate that the SAAL method can achieve regular and high-purity amorphous modification in an all-inorganic crystalline matrix with high efficiency, flexibility, and low energy. As shown in Fig. 3, multiple technologies have been applied to characterize and optimize the quality of SAAL-produced phase-transition units. Firstly, our holoview 3D refractive index imaging confirms that these units exhibit a highly regular, anisotropic, sheet-like spatial morphology in free space (Fig. 3a), consistent with the optical microscopic images in the XY and YZ planes (Fig. 3b, *left*). The significant refractive index contrast between amorphous LN and the crystalline matrix can produce significant phase modulation of O and E light propagating through the crystal. Consequently, an artificial birefringence signal can be observed near the phase-transition structure (Fig. 3b, *right*). Theoretically, the amorphization degree of the SAAL-modified region can be estimated by the intensity of the artificial birefringence. Experimental results have shown that the amorphization phase transition approaches completion (the birefringence signal closely approaches maximum) when the pulse width is greater than 1 ps and the pulse energy is greater than 0.55 μJ (Fig. S4). Further high-resolution transmission electron microscopy (HRTEM) characterization has confirmed that in this case, the SAAL-modified region has become completely amorphous, with a clear phase transition interface, which is in good agreement with the birefringence experiments (Fig. 3c).

Due to the inherent birefringence of LN crystals, a single ultrafast pulse can be split into an O pulse and an E pulse with different propagation velocities in the crystal. The E pulse, which arrives at the focal plane first, initially modifies the material, creating a large number of active defects. This significantly promotes the nonlinear optical absorption of the O pulse by the material in the irradiated area^19^, thus enabling SAAL with a single low-energy pulse. Experiments have shown that the pulse energy threshold of SAAL, based on the synergistic effect of the O and E pulses (synergistic mode), is an order of magnitude lower than that of the traditional non-synergistic mode (Fig. 3d). This effect allows SAAL to achieve high-purity amorphization with a single low-energy pulse, avoiding the formation of undesirable structural defects, such as microcracks. These defects are typically caused by multi-pulse high-energy injection in all-inorganic dielectric materials with rigid ionic or covalent bonds. This ensures superior processing quality in the resulting amorphous architectures (Fig. S5).

SAAL offers simple and efficient manipulation, easily achieved by constructing anisotropic optical fields. For example, a spatial light modulator, optical slits, and diffraction elements can be applied to transform a Gaussian spot into an anisotropic profile. As long as the laser parameters meet the requirements for high-density free electron excitation, SAAL can disregard the intrinsic thermal anisotropy of the crystal and produce amorphous units with arbitrary orientations (Fig. 3e). By simply using an optical slit, the length of the amorphous units can also be flexibly tuned from 2 to 38 μm (Fig. 3f). SAAL delivers considerably high fabrication precision, as confirmed by optical and scanning electron microscopy (SEM) characterizations. Cross-sectional SEM imaging in the XY plane reveals that amorphous units possess smooth boundaries and a consistent width of ~200 nm (Fig. 3g). A single Gaussian-typed pulse irradiation can produce an amorphous unit with an aspect ratio reaching 35:1. Using an anisotropic beam shaping, this value can be remarkably increased to 190:1 (Fig. 3h). The independently controllable orientation and aspect ratio of the amorphous units provide two additional degrees of freedom to construct complex 3D photonic architectures. It is worth noting that due to the nonlinear processing principle of ultrafast lasers, SAAL has no material dependence and can be widely extended to a variety of dielectric crystals, such as quartz crystals, lithium tantalate crystals (LiTaO_3_), yttrium orthovanadate (YVO_4_), and potassium titanyl phosphate crystals (Fig. 3i), showing its excellent cross-material universality. In addition, we also demonstrated that SAAL maintains considerable cross-material repeatability in massive production (Fig. S6 and Note S3).

**Fig. 4:**
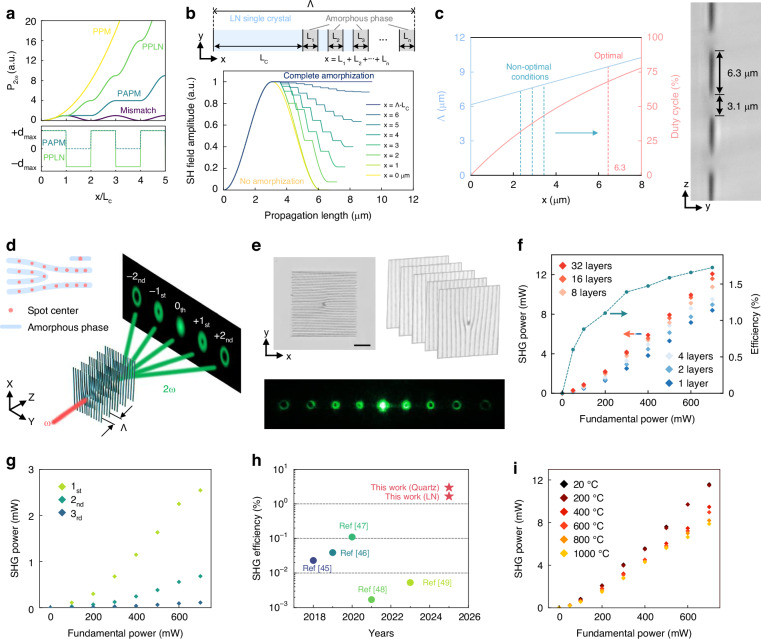
SAAL-based nonlinear light modulation in LN crystal. **a** Amplitude of SHG in different phase-matching modes, including perfect phase matching (PPM), periodically poled lithium niobate (PPLN), periodical amorphization-based phase matching (PAPM), and mismatch. *d*_max_ indicates the largest nonlinear coefficient. *L*_c_ is the coherence length. *x* is the length of the modified regions. **b** Non-optimal PAPM caused by incomplete amorphization (upper) and calculated SH field amplitude at different *x* values (lower). *L*_*n*_ indicates the length of amorphous regions. **c** Structural design principle for realizing high-efficiency PAPM (left) and an experimentally produced one (right). Λ indicates the period of amorphous regions. **d** SAAL-based production of embedded nonlinear photonic structures in dielectric crystals. ω is the light frequency. **e** Experimentally produced multi-layer fork-shaped grating structure (upper) and nonlinear beam shaping results (lower). Scale bar: 50 μm. **f** SHG power and efficiency as a function of fundamental power and layer number. **g** SHG power of vortex beams at different diffraction orders as a function of fundamental power. **h** SHG efficiency comparison of this work and previous nonlinear beam shaping approaches^45–49^. **i** Stability characterization of amorphous photonic structures by heat treatments at different temperatures

Quasi-phase matching (QPM) by engineering the second-order nonlinear coefficient χ^(2)^ has become an important way to achieve SHG in LN crystals (Fig. 4a), especially for constructing 3D integrated nonlinear photonic elements^24,44^. However, the multi-pulse modification of conventional laser direct writing approaches usually introduces more complex nonlinear ultrafast laser-matter interaction effects, leading to additional unfavorable micro-nanostructures, such as periodic interference patterns or nanostripes^4,31^. These impure phase transitions severely reduce the amorphization purity of the irradiated region, leading to imperfect phase matching in multiple forms (Fig. 4b), hindering full compensation of the phase mismatch between the fundamental wave and the harmonics, and ultimately limiting the maximum achievable SHG efficiency.

Benefiting from the single-pulse modification mechanism, SAAL-fabricated units exhibit considerable structural uniformity, enabling efficient light modulation within all-inorganic crystalline materials. Specifically, to optimize QPM through selective χ^(2)^ erasure, the period of the amorphous unit arrangement and the duty cycle of the amorphous region should satisfy a specific relationship (Fig. 4c, *left*). SAAL achieves this by producing high-purity amorphous units (χ^(2)^ equals zero) with much higher precision, closely approaching the theoretically optimal phase-matching condition (corresponding to the red dotted line) and maximizing the SHG efficiency (Fig. 4c, *right*). By strategically engineering the arrangement and combination of these highly regular phase-transition units, SAAL enables efficient fabrication of 3D nonlinear optical elements directly within crystalline matrices (Fig. 4d). As a proof of concept, we fabricated a multilayer fork-shaped grating structure using SAAL (with a footprint of only 200 × 200 μm) for simultaneous conversion of optical frequency and polarization state. This device generates and angularly separates second-harmonic (SH) vortex beams with distinct topological charges (Fig. 4e). By approaching the optimal phase-matching conditions, the aggregate conversion efficiency for collinear and non-collinear SH spots reaches ~1.7% (Fig. 4f), where the individual efficiencies for the first-, second-, and third-order SH vortex beams can reach 0.38%, 0.097%, and 0.016%, respectively (Fig. 4g), representing an order-of-magnitude improvement over the state-of-the-art methods (Fig. 4h). These amorphous photonic structures inherently possess excellent stability, further reinforced by the protection of the all-inorganic crystal matrix. We demonstrate that even after annealing at 1000 °C for 20 min, the aggregate conversion efficiency remains at 1.13% (Fig. 4i), and the thermal stability of the amorphous units also exhibits exceptional reproducibility (Fig. S7), highlighting the great potential of SAAL in constructing non-volatile photonic devices. By optimizing the laser parameters, the cross-sectional dimensions of this structure can be further miniaturized to 10 × 10 μm, facilitating 3D integration with embedded photonic elements (Fig. S8).

The excellent universality of SAAL allows its extension to various dielectric crystals, thereby maximizing the advantages of the outstanding optical properties of different matrix materials. Compared to LN, quartz crystal is not a ferroelectric crystal, lacking hysteresis loops and switchable spontaneous polarization. Therefore, traditional domain engineering based on ultrafast laser direct writing cannot achieve effective nonlinear optical modulation within quartz crystals. By contrast, almost all dielectric crystals can be amorphized, which means that SAAL processes can still be applied to create amorphous phase-transition photonic structures within quartz crystals. The key advantage of quartz crystal lies in its excellent transparency over a wide wavelength range, a property shared by its amorphous phase, enabling the realization of a variety of nonlinear optical modulations. More importantly, the refractive index difference between quartz and its amorphous phase is considerably small (~10^-3^ level). This effectively avoids scattering loss of the fundamental, frequency-doubled, and sum-frequency light, enabling higher frequency conversion efficiency.

**Fig. 5:**
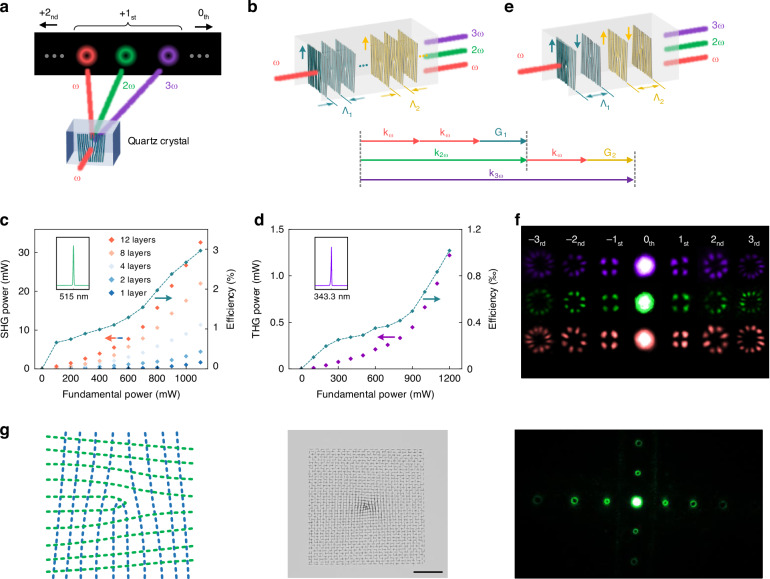
SAAL-based multi-functional nonlinear light modulation in quartz crystal. **a** Schematic diagram of multi-functional nonlinear beam shaping using SAAL-produced photonic architectures. **b** Phase-matching principle for simultaneous generation of SH and TH vortex light beams by constructing multi-layer fork-shaped gratings. k_ω_, k_2ω_, and k_3ω_ are the wavevectors of the fundamental, SH, and TH waves, respectively. G_1_ and G_2_ are reciprocal vectors. Λ_1_ and Λ_2_ are periods of the multi-layer gratings. **c** SHG power and efficiency as a function of fundamental power and layer number. Inset: spectrum of the SH beam. **d** THG power and efficiency as a function of fundamental power at a layer number of 15 (detailed structure design see “Methods”). Inset: spectrum of the TH beam. **e** Schematic diagram of periodically inverted cascaded fork-shaped gratings that can make the SH and the TH vortex beams with opposite topological charges interfere with each other, respectively. **f** Experimentally generated complex nonlinear light fields using the design presented in (**e**). **g** Design (left) and fabrication (middle) of a compound nonlinear grating with more complex interior structures and the SH vortex arrays (right) generated by the fabricated structure. Scale bar: 50 μm

As a proof of concept, we propose that multi-layer cascaded fork gratings can be engineered in a quartz crystal to simultaneously generate and output SH and third harmonic (TH) vortex beams (Fig. 5a). Specifically, by carefully designing the spatial distribution of periodic amorphous fork gratings structures, we sequentially arrange the QPM configurations for SHG and sum frequency generation (SFG). This allows the light generated via the SHG process to serve as one of the inputs for the subsequent SFG process. Thus, the third-harmonic generation (THG) can be enhanced through a cascaded process (2ω + ω = 3ω), with efficiency far exceeding that of the direct THG process (ω + ω + ω = 3ω). Then, the fundamental frequency light, the frequency-doubled light, and the sum frequency light are converted into vortex light through the fork grating and output separately (Fig. 5b). Experiments show that our nonlinear photonic structure can simultaneously generate and output SH and TH vortex light, with the SH conversion efficiency reaching 3% (Fig. 5c) and the TH vortex light conversion efficiency reaching 0.1% (Fig. 5d). Interestingly, the SHG efficiency achieved in quartz crystal is higher than that in LN crystal, despite quartz’s substantially smaller χ^(2)^. To the best of our knowledge, this is the most efficient nonlinear beam shaping achieved in a single quartz crystal. This enhancement is attributed to the close integration of different QPM configurations in the same medium, which eliminates additional optical alignment or multi-device splicing that introduces interface reflection, scattering, and phase discontinuities, thereby achieving much higher efficiency. On this basis, we can use the flexibility of SAAL to achieve more complex nonlinear beam shaping. For example, by preparing periodically inverted (with identical periods but opposite orientations) cascaded fork gratings (Fig. 5e), we can make the SH and the TH vortex beams with opposite topological charges interfere with each other, respectively, ultimately forming a series of Hermite-Gaussian beams in the far field (Fig. 5f). Additionally, leveraging the multi-dimensional controllability and straightforward process of SAAL, more complex 3D embedded amorphous architectures can be efficiently produced with high quality by simultaneously tuning the irradiation position and the optical slit orientation (Fig. S9). As a proof of concept, we demonstrate a multi-layer compound nonlinear grating that can achieve SH vortex beam arrays in two designed directions without any stray diffraction spots in other directions (Fig. 5g). These results highlight the potential of SAAL in achieving more efficient and complex nonlinear optical modulation even in weakly nonlinear materials.

## Discussion

Traditionally, achieving high-quality amorphization phase transitions in all-inorganic dielectric crystals has long been challenging. Current approaches (e.g., laser direct writing, laser-induced self-organizing) typically suffer from slow processing speed, limited structural regularity, and low optical modulation efficiency caused by phase impurity. In this study, we reveal the crucial role of high-density free electrons in ultrafast laser-induced anisotropic thermal deposition and propose a novel phase-transition lithography method. This technique requires only a single ultrafast laser pulse to induce high-purity amorphous units within dielectric crystals in one step. These units achieve feature sizes as small as 200 nm and aspect ratios up to a 200:1 level, enabling flexible manipulation via multiple approaches. Furthermore, the method exhibits exceptional universality across diverse dielectric crystal materials. We leverage these amorphous units to rapidly fabricate 3D multilayer cascaded photonic structures within key nonlinear dielectric crystals. These structures demonstrate nonlinear optical modulation in high-refractive-index dielectric environments, including SHG, THG, and the generation of optical vortices with varying orbital angular momentum and energy distributions. The resulting conversion efficiency significantly surpasses that achieved by existing similar approaches. Encapsulated within an all-inorganic crystalline matrix, these photonic devices exhibit exceptional robustness and withstand various extreme environmental disturbances. Their compact size enables direct integration with a wide range of established photonic elements to achieve multi-functional systems.

In summary, this work achieves a significant advancement in the precise inscription of high-purity phase-transition micro-nanostructures within inorganic transparent dielectrics. The proposed structuring method, energy deposition mechanism, and created amorphous photonic architectures offer a versatile and accessible approach that unlocks new opportunities for enhancing nonlinear light-matter interactions in transparent dielectrics and for advancing free-space integrated optical devices. Future developments could integrate this technique with advanced optical modulation, topology design, and metamaterials engineering to establish a highly versatile, all-inorganic dielectric lithography platform, potentially paving the way for the efficient realization of next-generation 3D in-chip photonic systems.

## Methods

### Fabrication of 3D photonic architectures

An ultrafast laser direct writing system is utilized for SAAL (Fig. S10). The light source comprises a mode-locked, regeneratively amplified Yb:KGW-based ultrafast laser (PHAROS, Light Conversion Ltd.) operating at a wavelength of 1030 nm, with pulse durations ranging from 226 fs to 6 ps. Laser power is regulated via an attenuator (LBTEK). The laser beam is focused into crystals through a 50× objective lens with a numerical aperture of 0.8. All crystals used in this study are commercially available standard optical crystals. Laser parameters for processing three-dimensional nonlinear photonic crystals should ensure that the size of amorphous region satisfies the QPM condition along the Z-direction, and a rotatable slit with a width of 500 μm is used to facilitate the flexible construction of 3D photonic architectures. Typically, multi-layer fork gratings in LN crystals are fabricated via a single pulse ultrafast laser with pulse duration of 2 ps and pulse energy of 440 nJ. The multi-layer fork gratings are produced layer by layer, progressing from deep to shallow within the crystals, with the initial depth set at 400 μm below the surface of LN crystal. The period between adjacent layers is 9.4 μm, and the size of the amorphous unit in the Z-direction is 6.3 μm. In quartz crystals, fork gratings are fabricated using a single pulse ultrafast laser with pulse duration of 1 ps. The pulse energies are set to 8.8 μJ and 1.47 μJ for the creation of a cascaded fork grating with periods of 38.9 μm and 14.5 μm for SFG. The initial depth is set at 525 μm below the surface of the quartz crystal. Further scaling of multilayer photonic structures is related to the crystal thickness. For instance, according to the requirements of QPM for the duty cycle and periodicity, theoretically, up to 50 layers of amorphous phase transition structures can be fabricated in a 500 µm thick LN crystal.

### Nonlinear beam shaping measurement

A linear polarized laser source with a central wavelength of 1030 nm and a pulse duration of 226 fs is employed as the fundamental frequency light for SHG and THG. The LN and quartz crystals used in SHG and THG are Y-cut and Z-cut, respectively. The incident Gaussian beam is first polarized with a Glan prism and then adjusted by a half-wave plate to make the polarization direction of the fundamental frequency light aligned with the c-axis of LN and the a-axis of quartz crystals to maximize their effective nonlinear coefficients d_33_ and d_11_. The laser is focused onto the 3D photonic architectures within the crystal through a biconvex lens with a focal length of 100 mm. The far-field pattern is projected onto a white screen and captured by a camera.

### SEM characterization

The amorphous structure was imaged using a field-emission scanning electron microscope (GeminiSEM 300, ZEISS) in backscattered-electron mode, operated at an accelerating voltage of 15 kV and a working distance of 14.7 mm. For sample preparation, the laser-machined amorphous structure of LN was metallographically polished to expose it at the surface.

### TEM characterization

TEM lamellae were prepared by focused ion beam (FIB) milling using a FEI Helios NanoLab G3 UC. Initial thinning employed a 30 kV Ga⁺ beam. To reduce ion-beam damage, further thinning was performed at 5 kV and 1 kV with beam currents decreasing from 15 to 5 pA, followed by a final surface clean at 0.5 kV. TEM imaging was carried out on an FEI Tecnai F20 operated at 200 kV.

## Supplementary information

Supplementary note and figure

## Data Availability

All data needed to evaluate the conclusions in the paper are available in the main text or the supplementary materials.
