# Supplementary material for: Single-pulse lithography of amorphous photonic architectures inside all-inorganic dielectric crystals
Source: Light Sci Appl. 2026 Mar 18;15:177. doi: 10.1038/s41377-026-02253-1 (PMC12996397; doi:10.1038/s41377-026-02253-1)
Supplement: Supplementary file 1 — Supplementary note and figure [file 41377_2026_2253_MOESM1_ESM.docx]

Supplementary Information for

**Single-pulse lithography of amorphous photonic architectures inside all-inorganic dielectric crystals**

*Zhuo Wang^1†^, Rongze Ma^1†^, Han Lin*^2^*,* *Pengfei Zhang*^3^*, Yu Lu*^3^*, Feng Chen*^3^*,*

*Baohua Jia*^2^*, Bo Zhang^1*^, Jianrong Qiu^1*^*

^1^State Key Laboratory of Extreme Photonics and Instrumentation, College of Optical Science and Engineering, Zhejiang University, Hangzhou 310027, China

^2^Centre for Atomaterials and Nanomanufacturing, School of Science, RMIT University, Melbourne 3000, VIC, Australia

^3^State Key Laboratory for Manufacturing System Engineering and Shaanxi Key Laboratory of Photonics Technology for Information, School of Electronic Science and Engineering, Xi'an Jiaotong University, Xi'an, 710049, China

*Corresponding author Email: zhangbobo@zju.edu.cn; qjr@zju.edu.cn

†These authors contributed equally to this work

### Supplementary Note

**Note S1: Two-temperature model**

When ultrafast lasers interact with matter, the timescale of photo-induced electron excitation is significantly earlier than that of electron-phonon interaction. Therefore, when the temperature of electrons reaches a high level, the temperature of the lattice remains very low, which is a typical non-equilibrium process. S.I. Anisimov et al. proposed the two-temperature model (TTM) in 1974^1^. This model takes into account two different interaction processes between ultrashort pulses and electrons and between electrons and the lattice and describes the temperature change process of electrons and the lattice through two differential equations, respectively. Although this model was initially widely applied to discuss various dynamic phenomena of ultrafast laser ablation of metals, its core idea did not limit it to metallic materials. In 2002, E.G. Gamaly et al. revealed through a large number of analytical calculations that under the irradiation of high-intensity (10^14^ W∙cm^-2^) ultrafast lasers, the material ionization is completed before the end of the pulse action time. At this point, the modification mechanisms of ultrafast laser processing become consistent for both metals and dielectrics^2^. To date, numerous studies have shown that the TTM can be extended to various dielectric materials while maintaining considerable reliability.

The temperature distribution during single-pulse ultrafast laser-driven anisotropic amorphization lithography (SAAL) in lithium niobate (LiNbO_3_) crystals is simulated based on the TTM, which characterizes the temperature evolution of both electronic and lattice subsystems through coupled partial differential equations^3^:

$C_{e}\frac{\partial T_{e}(r,t)}{\partial t}=\nabla\left( k_{e}\nabla T_{e} \right)-G(T_{e}-T_{l})+S(r,t)$ (S1)

$C_{l}\frac{\partial T_{l}(r,t)}{\partial t}=\nabla\left( k_{l}\nabla T_{l} \right)+G(T_{e}-T_{l})$ (S2)

where $C_{e}=3K_{b}(\frac{n_{e}}{2})$ and $C_{l}=\rho c_{p}$, corresponding to electron heat capacity and lattice heat capacity, respectively. Here, $K_{b}$ is the Boltzmann constant, $n_{e}$ is the free electron density, $\rho$ is the density of crystal, $c_{p}$ is specific heat capacity of unit volume in lattice system. $T_{e}(r,t)$ and $T_{l}(r,t)$ represent electron temperature and lattice temperature, which are spatiotemporal functions. $k_{e}$ and $k_{l}$are electron thermal conductivity and lattice thermal conductivity, $G$ is the electron–phonon coupling coefficient, and $S(r,t)$ is the laser source term.

The free electron density ($n_{e}$) excited by ultrafast laser is contributed with multiphoton ionization, avalanche ionization, diffusive loss, recombination, and depth-wise diffusion, which can be theoretically estimated by solving the following rate equation^4,5^:

$\frac{\partial n_{e}}{\partial t}=\left( \frac{\partial n_{e}}{\partial t} \right)_{m}+\eta_{a}n_{e}-{gn}_{e}-\eta_{r}{n_{e}}^{2}$ (S3)

where $\left( \frac{\partial n_{e}}{\partial t} \right)_{m}$, $\eta_{a}$, $g$, and $\eta_{r}$ represent photoionization rate, avalanche ionization rate, diffusion rate and recombination rate.. The multiphoton ionization rate can be calculated based on Keldysh's model^6^. The avalanche ionization rate is related to the energy gain of the electrons from the electric field and the energy transfer from the electrons to the heavy molecules during elastic collisions. The electron diffusion rate is affected by the band gap energy of crystal and the spatial propagation property (i.e. Rayleigh length and beam waist) of employed ultrafast laser^7^. The recombination rate is a constant measured by Docchio^8^.

**Note S2: Thermal flux evaluation**

Since the system dimension of ultrafast laser-matter interaction (micrometer scale) is much larger than the mean free path of electrons (nanometer scale), the thermal transport of high-density electrons is primarily accomplished through diffusion transport mechanisms. Under this mechanism, the thermal flux (*q*) that electrons can transport can be described as^9^:

$\text{q=-k}\frac{\text{∆T}}{\text{∆x}}$ (S4)

where *k* is the electronic thermal conductivity, $\frac{\text{∆T}}{\text{∆x}}$ is the temperature gradient, and the negative sign indicates that heat flows from high to low temperature. The SAAL mechanism is based on ultrafast laser-induced transient metallic states, with the excited electron density reaching 10^28^ m^-3^ and the electron temperature reaching 10⁷ K. According to the Wiedemann-Franz law and two-temperature model, the electronic thermal conductivity generally exceeds 100 W·m^-1^·K^-1^ (up to 10^6^ W·m^-1^·K^-1^) throughout the high-electron-density period. It is worth noting that the ultrafast laser-matter interaction process is a typical nonlinear and nonequilibrium process. Under the irradiation of a Gaussian beam, high-density free electrons are preferentially excited at the center of the laser focal region and absorb photon energy and reach extremely high temperatures (exceeding 10^6^ K before the lattice heating), while the electron density at the periphery of the focal region remains very low and negligible. Therefore, a significant temperature difference emerges between the center and periphery of the focal region (micron-scale spatial domain), yielding a considerably large temperature gradient (~10^11^ K·m^-1^). Based on these results, we preliminarily estimate that the electron’s thermal flux can reach at least ~10^13^ W·m^-2^ level within the ultrafast laser focusing region. These results well illustrate the feasibility of long-range thermal transport of high-density electrons.

**Note S3: Reproducibility and stability**

We have experimentally demonstrated the thermal stability of the amorphous units and the reproducibility of the SAAL technique across different crystals and experimental runs. Specifically, we performed 500 SAAL processing cycles in several important optical crystals, including LiTaO_3_, KTP, and YVO_4_ (**Fig. S6**). The resulting amorphous units exhibited highly consistent lengths across all materials. Specifically, the standard deviation was below 0.05 (variance ≈ 0.002) in LiNbO_3_, LiTaO_3_, and quartz, while in YVO_4_ and KTP it was approximately 0.14 (variance ≈ 0.02), demonstrating excellent reproducibility across different material systems. Furthermore, we demonstrated the high-temperature stability of the amorphous units in 100 lithium niobate (LiNbO_3_) samples. The experiments showed that all samples maintained high and consistent second-harmonic generation (SHG) efficiency after annealing at 1000 °C, with efficiency variations among different samples within ±0.1% (variance ≈ 0.001), confirming reproducibility under diverse experimental conditions. Similar thermal stability of the amorphous units was also confirmed in 100 quartz crystal samples, attesting to the cross-material reproducibility of this property (**Fig. S7**).

### Supplementary Figure


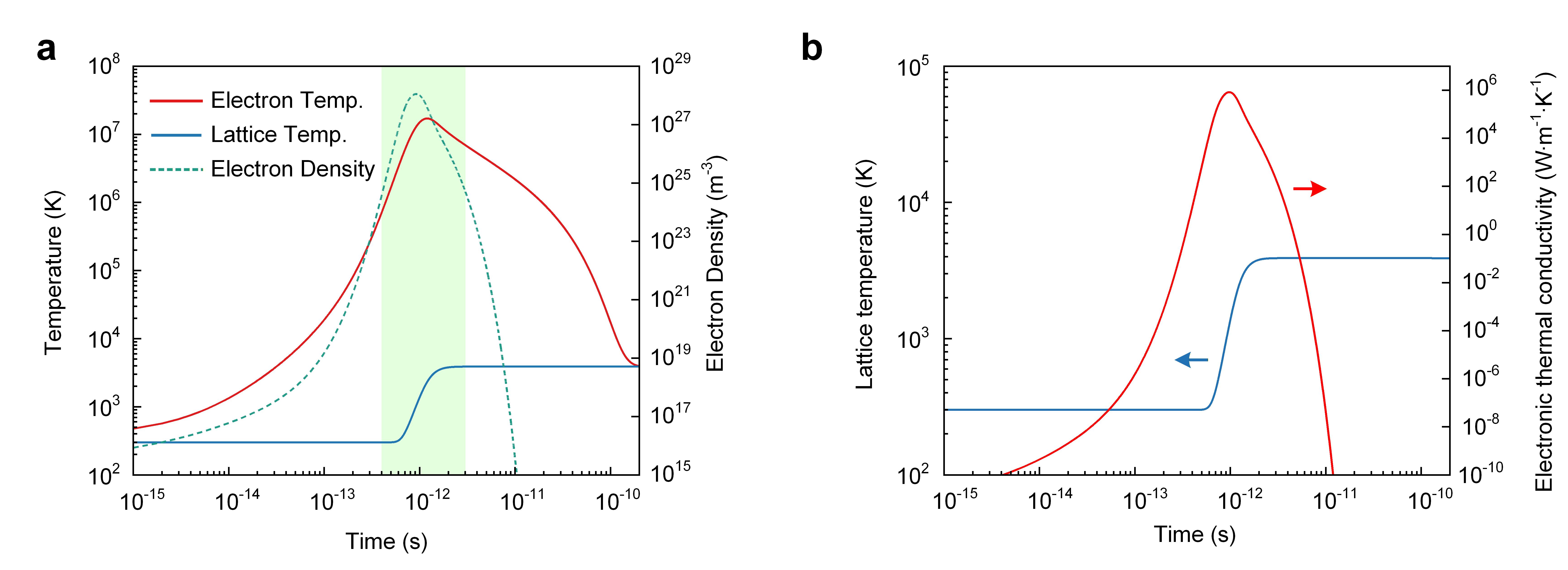


**Fig. S1** Theoretical calculation of system temperatures and electron thermal conductivity. **a** Temporal evolution of electron temperature, lattice temperature, and electron density in LiNbO_3_ crystal irradiated by a single-pulse ultrafast laser with a pulse duration of 600 fs and a pulse energy of 180 nJ. **b** Temporal evolution of electron thermal conductivity along with the lattice temperature.


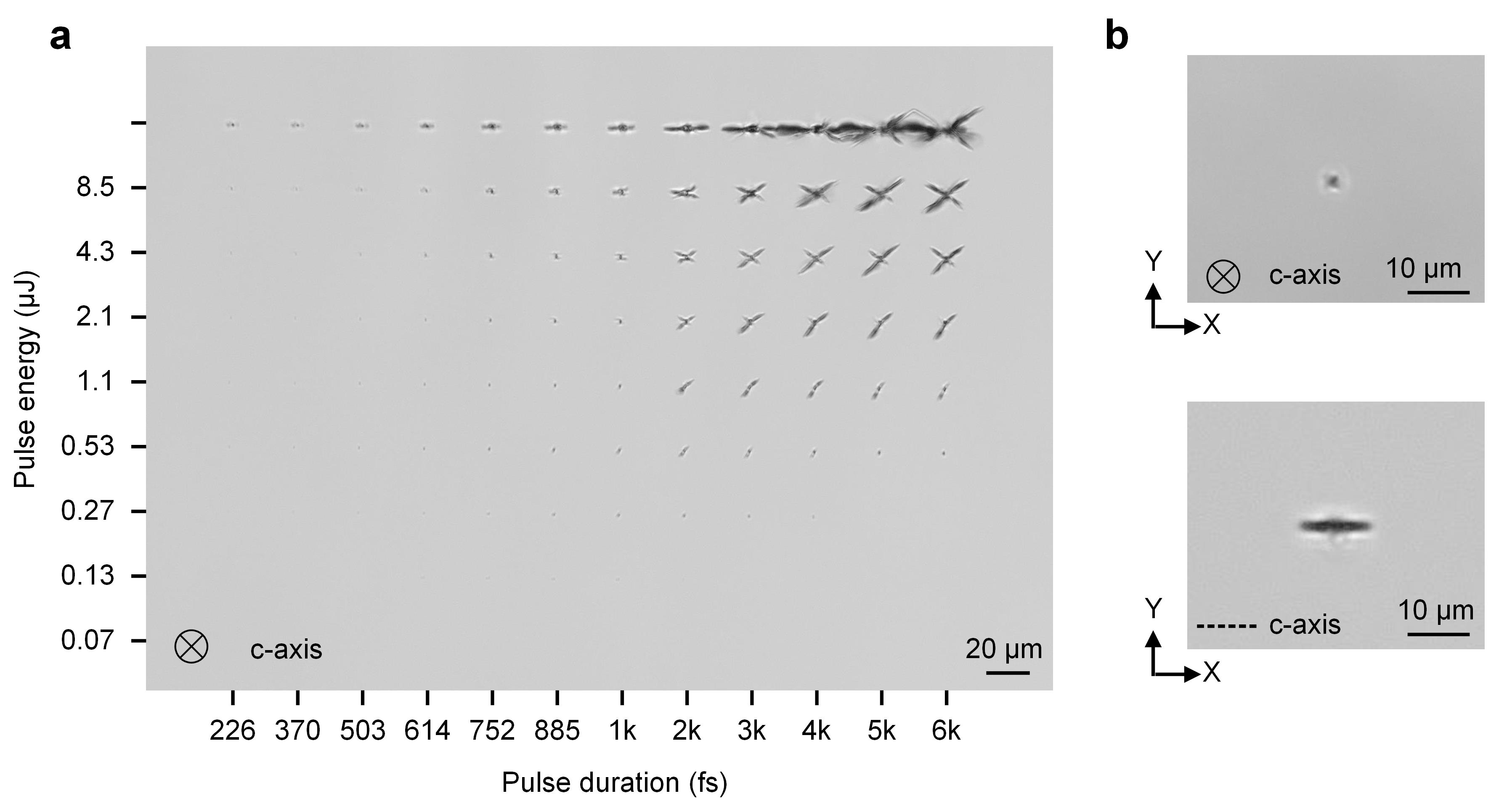


**Fig. S2** Effect of intrinsic thermal anisotropy of the matrix material on SAAL. a Structures induced by a Gaussian-profile laser spot with different pulse energies and pulse durations in Z-cut LiNbO_3_ crystal. b Structures produced in Z-cut and Y-cut LiNbO_3_ crystals using the same processing parameters.


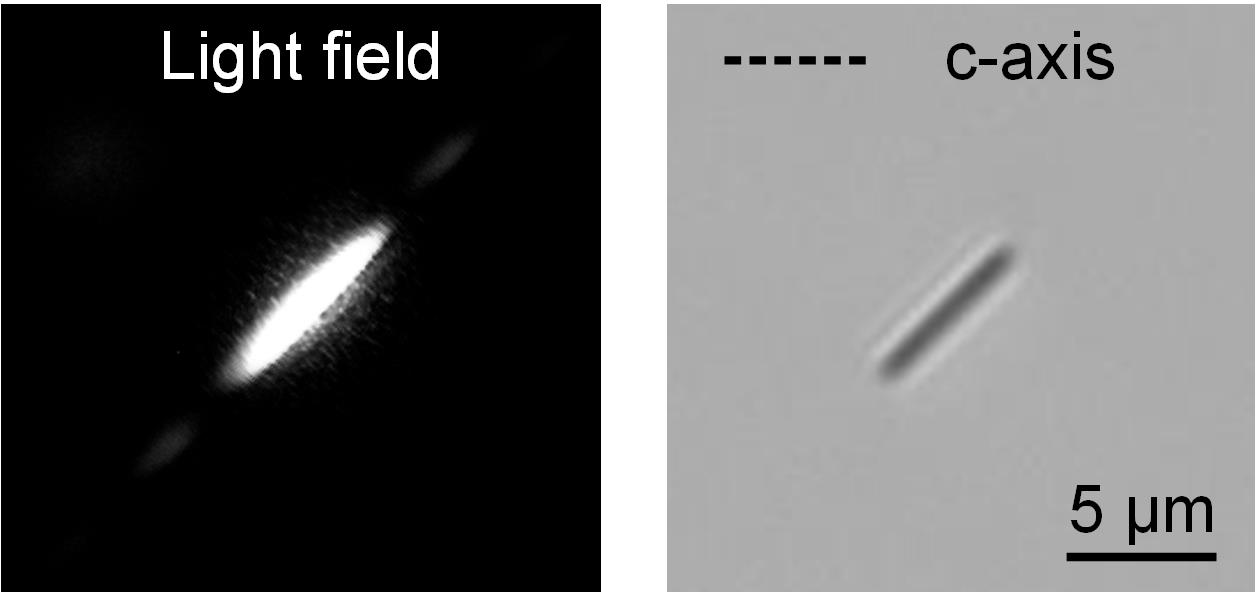


**Fig. S3** Optical image of anisotropic light field and SAAL-produced structure under the anisotropic light field.


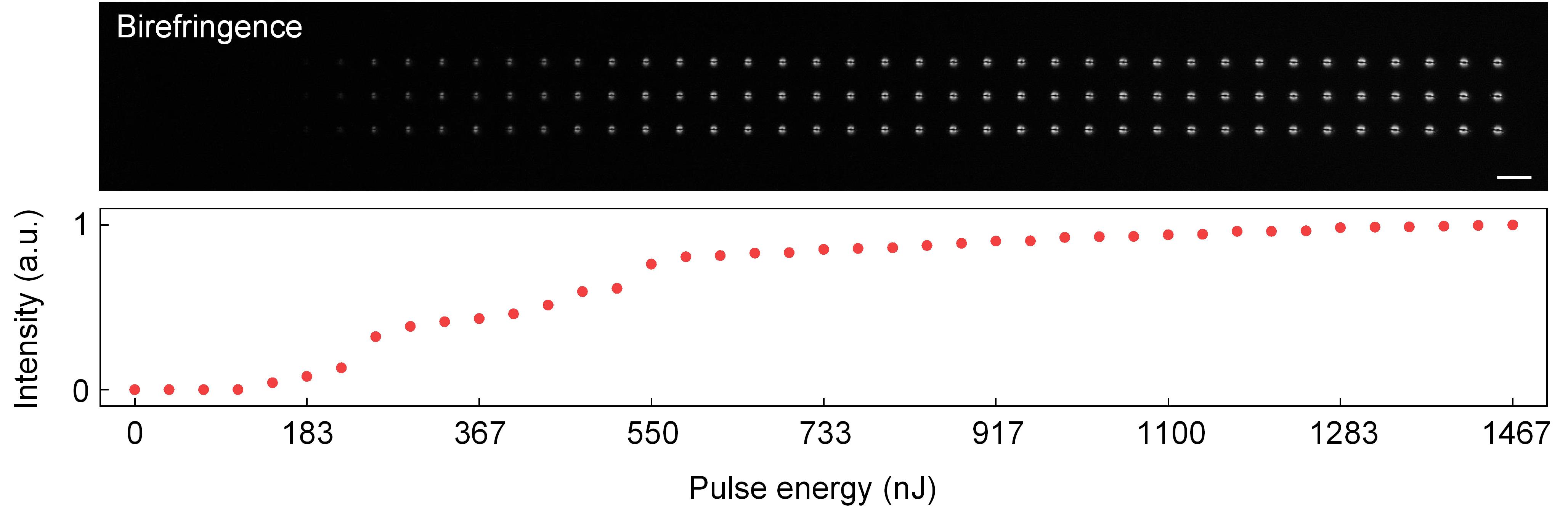


**Fig. S4** Birefringence signals of laser-induced amorphous structures with various pulse energies. Scale bar: 30 μm.


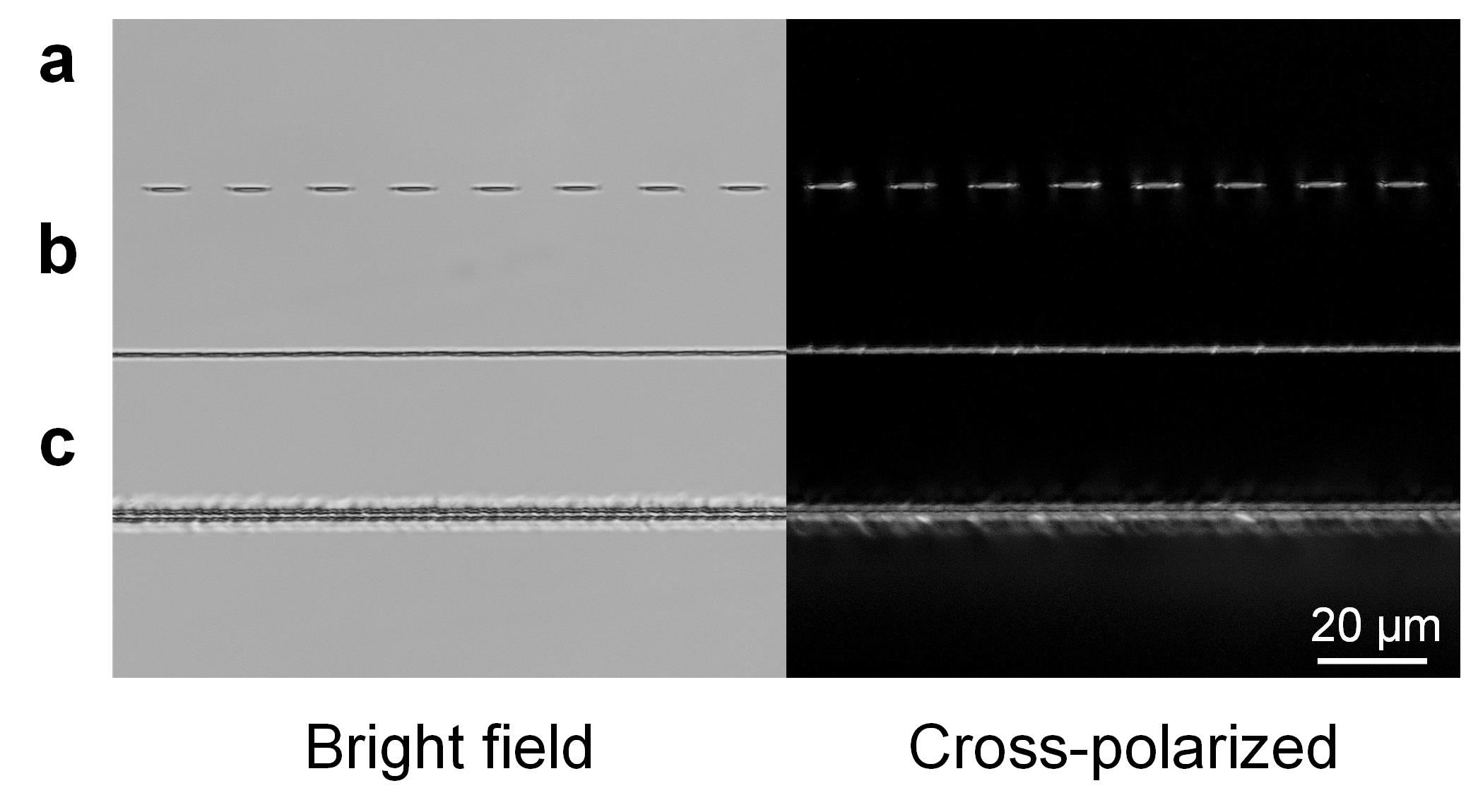


**Fig. S5** Structures induced by SAAL method and ultrafast laser direct writing. **a** SAAL-produced amorphous units in quartz crystal. **b** SAAL-produced continuous structure in quartz crystal. **c** Ultrafast laser direct writing-produced structures in quartz crystal. Pulse energy: 1.15 μJ.


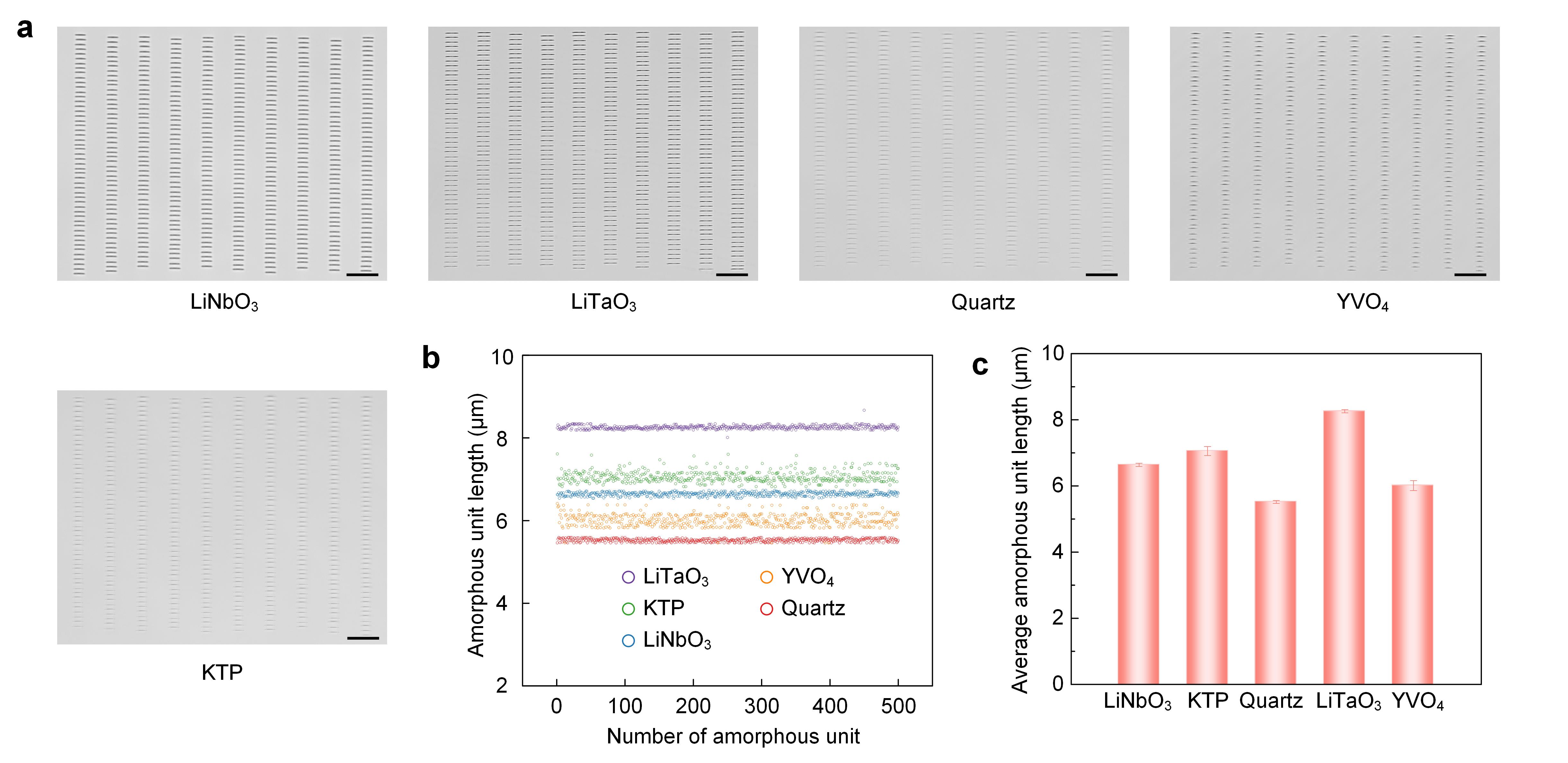


**Fig. S6** Repeatability of the SAAL process. **a** Optical image of numerous amorphous unit in different crystals. **b** Statistical data of these amorphous unit length in different crystals. **c** The average amorphous unit length in different crystals. Error bars represent standard deviation. Scale bars: 20 μm.


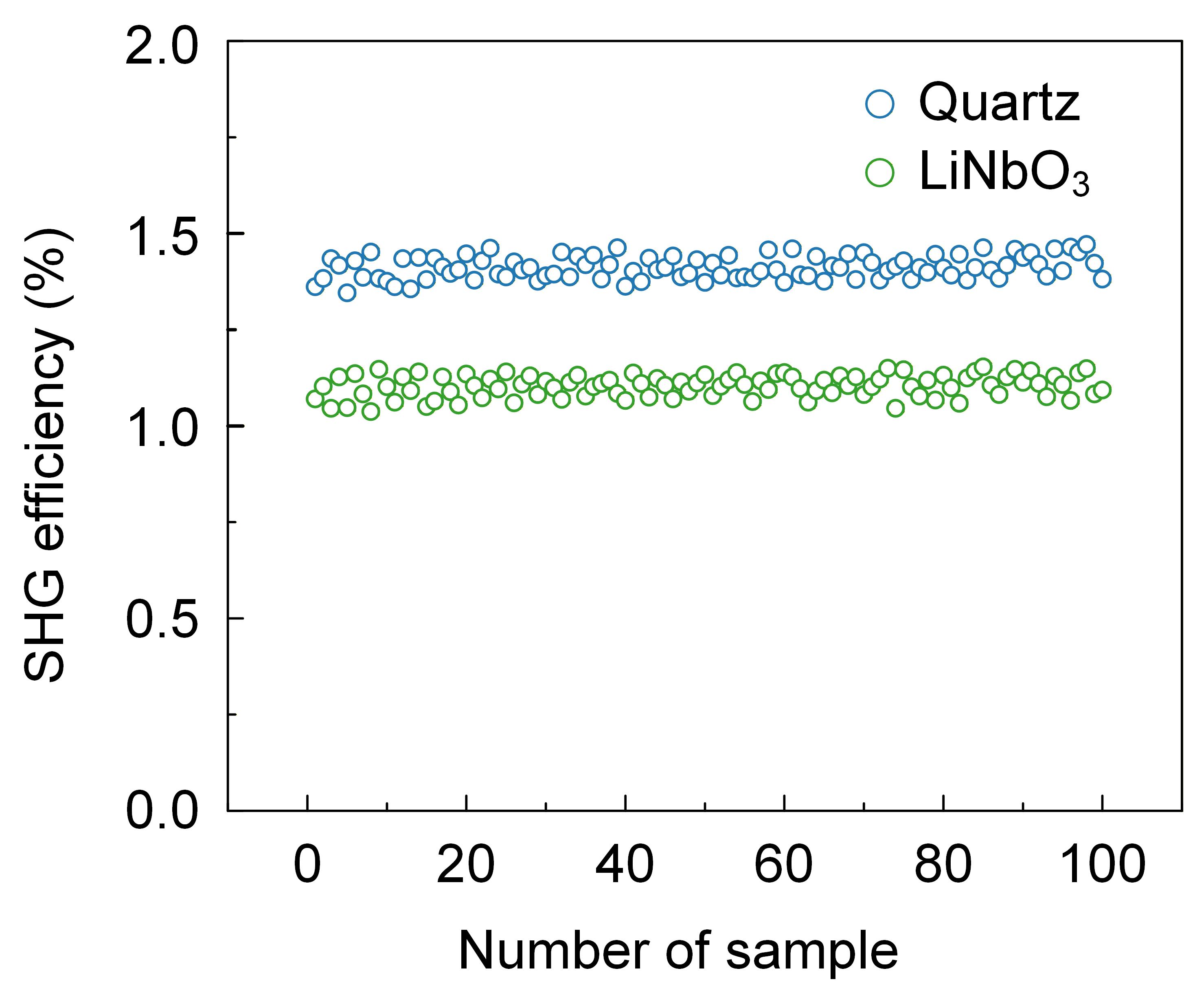


**Fig. S7** Thermal stability of amorphous photonic structures in 100 LiNbO_3_ samples and 100 quartz samples by heat treatments at 1000 °C.


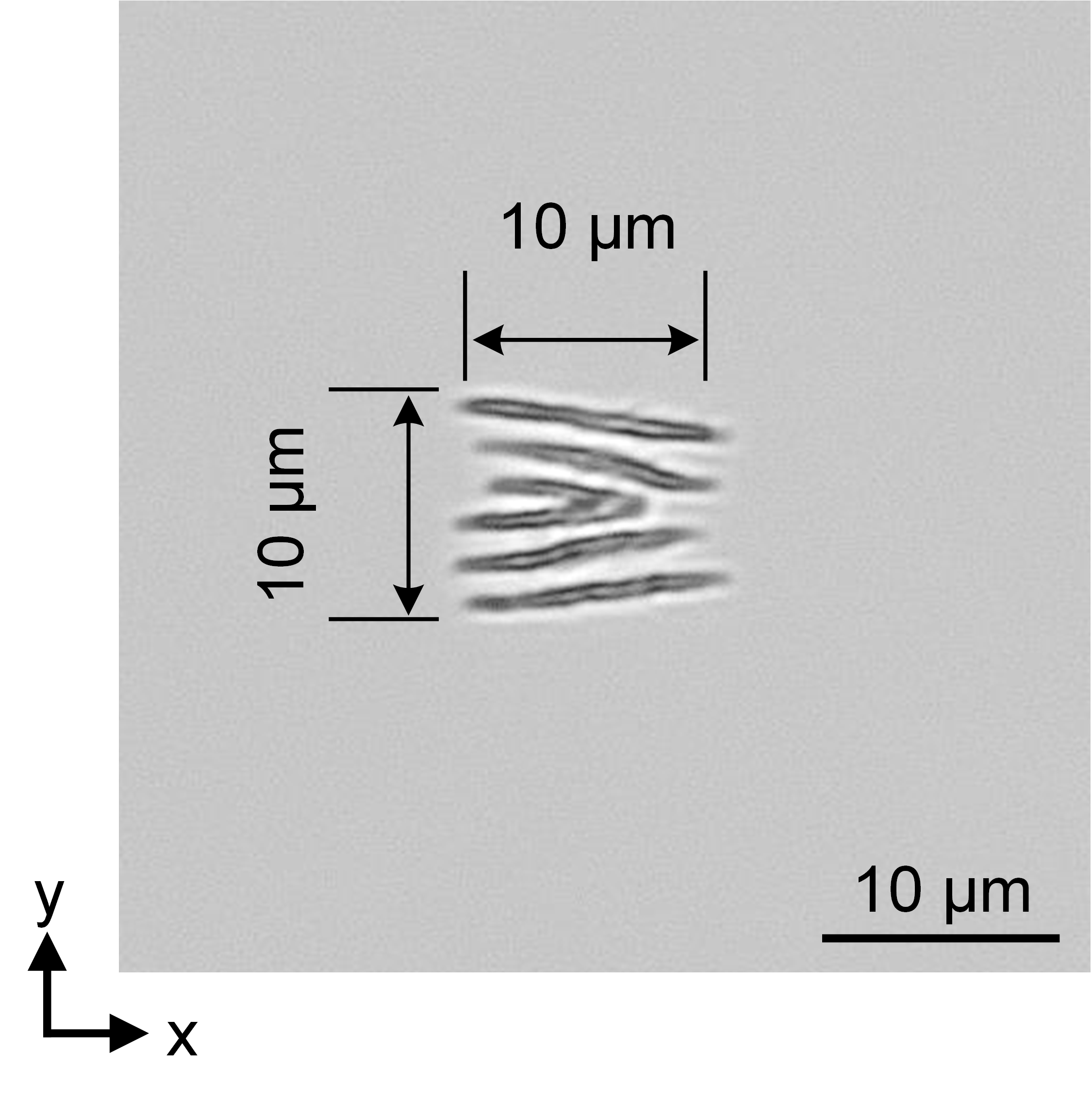


**Fig. S8** Optical image of the minimum fork-shaped grating.


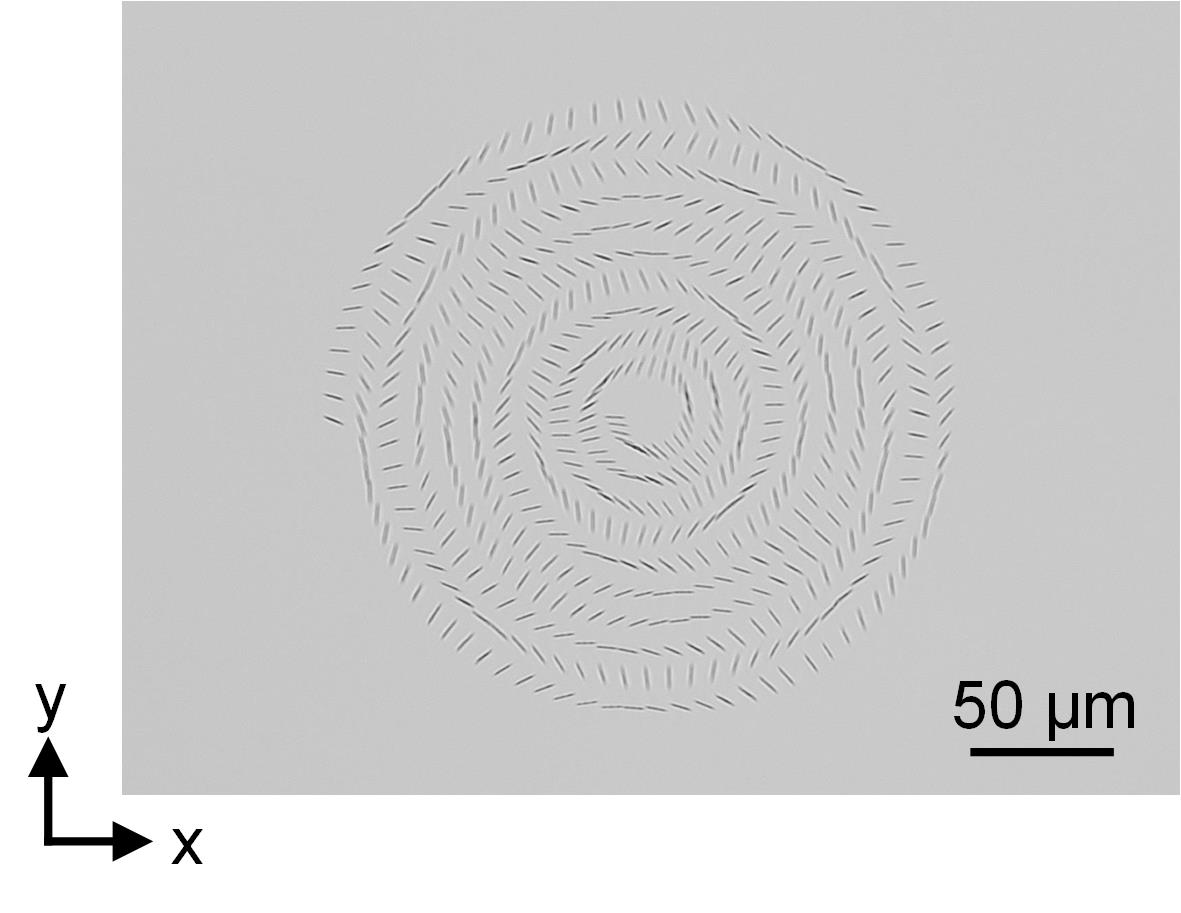


**Fig. S9** SAAL fabrication of more complex amorphous unit arrays by simultaneously tuning the irradiation position and the optical slit orientation.


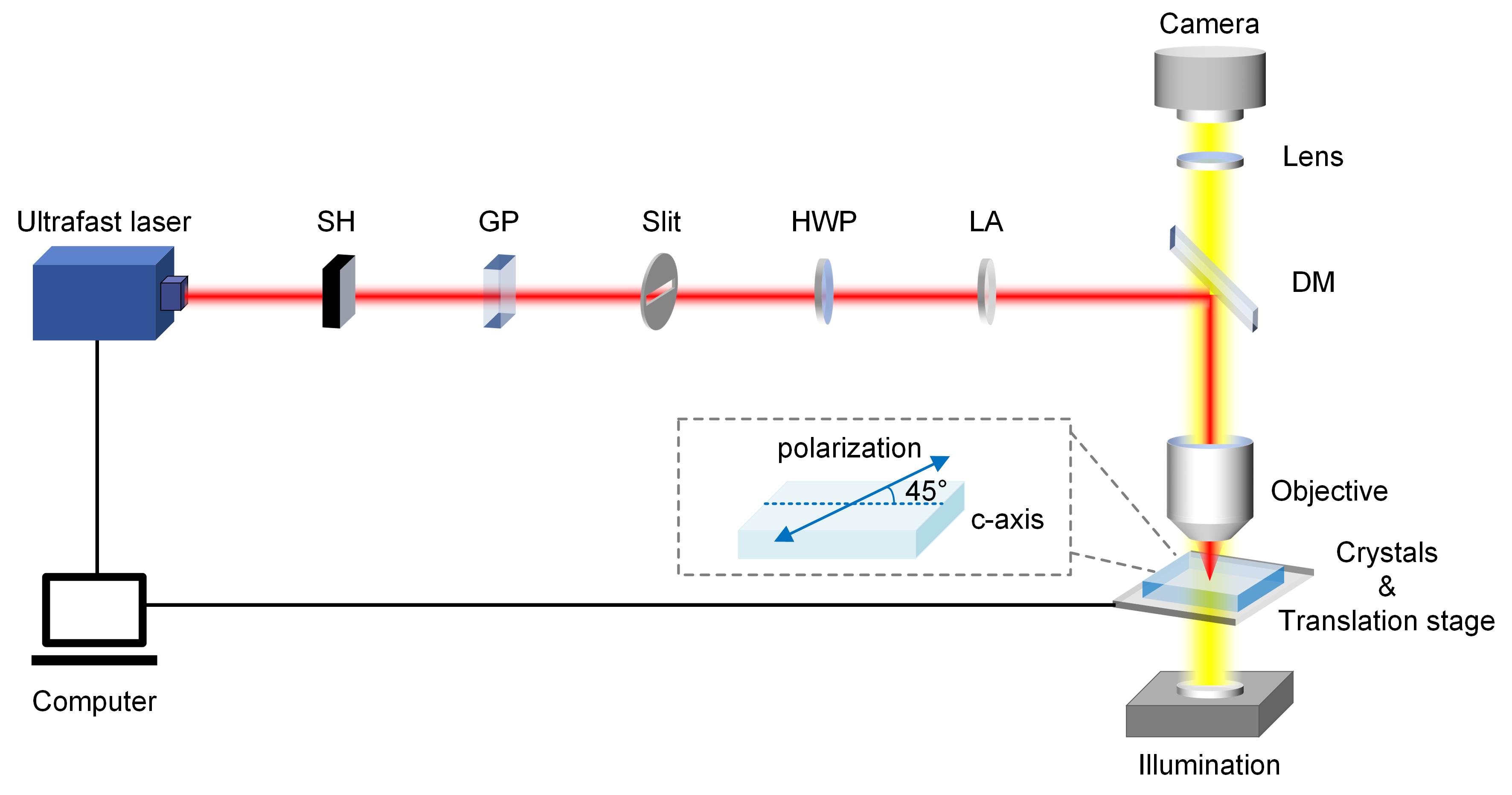


**Fig. S10** Experimental setup for laser processing. SH: shutter. GP: Glan prism. HWP: half wave plate. LA: light attenuator. DM: dichroic mirror.

### Supplementary Reference

1 Anisimov, S., Kapeliovich, B. & Perelman, T. Electron emission from metal surfaces exposed to ultrashort laser pulses. *Zh. Eksp. Teor. Fiz* **66**, 375-377 (1974).

2 Gamaly, E. G., Rode, A. V., Luther-Davies, B. & Tikhonchuk, V. T. Ablation of solids by femtosecond lasers: Ablation mechanism and ablation thresholds for metals and dielectrics. *Physics of plasmas* **9**, 949-957 (2002).

3 Su, Z., Meng, Q. & Zhang, B. Analysis on the damage threshold of MgO:LiNbO3 crystals under multiple femtosecond laser pulses. *Optical Materials* **60**, 443-449, doi:<https://doi.org/10.1016/j.optmat.2016.08.036> (2016).

4 Du, D., Liu, X., Korn, G., Squier, J. & Mourou, G. Laser‐induced breakdown by impact ionization in SiO2 with pulse widths from 7 ns to 150 fs. *Applied physics Letters* **64**, 3071-3073 (1994).

5 Sudrie, L. *et al.* Femtosecond Laser-Induced Damage and Filamentary Propagation in Fused Silica. *Physical Review Letters* **89**, 186601, doi:10.1103/PhysRevLett.89.186601 (2002).

6 Keldysh, L. Ionization in the field of a strong electromagnetic wave. *Sov. Phys. JETP* **20**, 1307-1314 (1965).

7 Noack, J. & Vogel, A. Laser-induced plasma formation in water at nanosecond to femtosecond time scales: calculation of thresholds, absorption coefficients, and energy density. *IEEE journal of quantum electronics* **35**, 1156-1167 (2002).

8 Docchio, F. Lifetimes of plasmas induced in liquids and ocular media by single Nd: YAG laser pulses of different duration. *Europhysics Letters* **6**, 407 (1988).

9 Incropera, F. P., DeWitt, D. P., Bergman, T. L. & Lavine, A. S. *Fundamentals of heat and mass transfer*. Vol. 1072 (New York John Wiley & Sons, Inc., 1990).
